# Patients' motives for choosing a physician: comparison between conventional and complementary medicine in Swiss primary care

**DOI:** 10.1186/1472-6882-7-41

**Published:** 2007-12-18

**Authors:** Victoria Wapf, André Busato

**Affiliations:** 1 Institute for Evaluative Research in Orthopedic Surgery, MEM Centre, University of Bern, Stauffacherstrasse 78, Bern, Switzerland

## Abstract

**Background:**

The study is part of a nationwide evaluation of complementary and alternative medicine (CAM) in primary care in Switzerland. The Objective was to identify patients' expectations and reasons governing the choice of complementary medicine compared with conventional primary care (CONV).

**Methods:**

The data were derived from the PEK study (Programm Evaluation Komplementärmedizin), which was conducted in 2002–2003 with 7879 adult patients and parents of 1291 underage patients, seeking either complementary (CAM) or conventional (CONV) primary care. The study was performed as a cross-sectional survey. The respondents were asked to document their (or their children's) self-perceived health status, reasons governing their choice, and treatment expectations. Physicians were practicing conventional medicine and/or complementary methods (homeopathy, anthroposophic medicine, neural therapy, and traditional Chinese medicine). Reasons governing the choice of physician were evaluated on the basis of a three-part classification (physician-related, procedure-related, and pragmatic/other reasons)

**Results and Discussion:**

Patients seeing CAM physicians tend to be younger and more often female. CAM patients referred to procedure-related reasons more frequently, whereas pragmatic reasons dominated among CONV patients. CAM respondents expected fewer adverse side effects compared to conventional care patients.

**Conclusion:**

The majority of alternative medicine users appear to have chosen CAM mainly because they wish to undergo a certain procedure; additional reasons include desire for more comprehensive treatment, and expectation of fewer side-effects.

## Background

Interest in and utilization of complementary and alternative medicine continue to grow in developing countries, including the USA [1-4]. Understanding the attractiveness of CAM is therefore crucial for providing better service in primary health care. The reasons of choice of patients for complementary medicine are based on both rational and emotional factors[5]. On one hand, those dissatisfied with orthodox medical treatment (who tend to cite impersonal service, low cost efficiency or general mistrust) turn to alternative medicine[1]. Others, in contrast, do not express such disappointment, but rather view CAM as supplementary measures in order to achieve the best possible results for their health [6].

The growing popularity of CAM methods might be explained by postmaterialistic trends that place individual perspectives ahead of scientific rationalism, and holistic interpretative models of health and disease, the "new age" values[7], which contradict conventional biomedical concepts and embrace a holistic approach based on a bio-psycho-social model. Though it is not possible to describe all users of CAM as a sole homogeneous group[8], the reasons to chose CAM may be influenced by socio-ethno-demographic attributes of populations and/or the nature of disease [9-11]. For instance, some Swiss studies show that there were more often female, with higher education, from upper middle class and aged between 30 and 50 among the CAM-users[6,12]. This group generally tends to have less children, thus it remains unclear whether the subgroup of parents of underage patients would match the profile of a typical Swiss CAM-user in terms of choice of treatment. However, motives and reasons to choose a particular physicians may affect the extent of utilization of health related resources That is why we have chosen to examine whether the preferences of both adult patients and parents acting on behalf of minors differ with respect to complementary or conventional medicine Following a political discussion, the Swiss Federal Department of Home Affairs decided in 1998 to add five methods of complementary medicine to the benefit package of basic health insurance for a period of five years. The methods included homeopathy, anthroposophic medicine, neural therapy, herbal medicine, and traditional Chinese herbal medicine. Because of the provisional status of coverage for CAM procedures in the health plans, a nationwide evaluation of CAM including several studies was performed [13]. As part of this evaluation, our study focused on the individual reasons and motives, and main health problems for seeking complementary and conventional care. We also examined whether the preferences of adult patients and parents acting on behalf of minors differ with respect to complementary or conventional medicine.

## Methods

### Design

Participating CAM physicians were selected from membership lists of societies for complementary medicine (Swiss medical associations for homeopathy, anthroposophic medicine, neural therapy, and traditional Chinese medicine) were obtained, and all CAM-certified physicians working as primary care physicians were asked to participate in the project. A list of all primary care providers (i.e. GPs, general internists) in Switzerland was additionally obtained from the Swiss Medical Association (FMH), from which a random sample of primary care providers not certified in any CAM discipline was selected and asked to participate. It was assumed that these physicians were less motivated to participate in the project. Therefore 1.5 times more non-CAM-certified physicians were sampled. This sample was proportionally matched to the regional distribution of physicians certified in complementary medicine.

The eligibility criteria for participating physicians required training and license to practice as a medical doctor in conventional medicine, medical activity in primary care for at least two days per week, and having at least five documented consultations within the study. For practitioners in alternative medicine, an additional qualification (recognized by the Swiss Medical Association, FMH) in one or more specific CAM disciplines was required. Physicians peforming CAM procedures without a corresponding certification were excluded from the study. Physicians were therefore classified into two categories based on their own declaration about their use of CAM and on the legal framework of reimbursing complementary medical services in primary care during the time of the study:

• Providers of conventional primary care only (CONV group)

• Providers of both conventional and complementary care, with additional professional certification in CAM (CAM group)

As for the patients, there were two inclusion criteria: willingness to participate, and ability to read and write in German, French, or Italian. Patients within the CONV and the CAM group respectively were further classified into two sub-groups:

• Adult patients over 16 years of age

• Minor patients (children) under 16 years of age, whose parents responded to the survey

Additional information about the scope and design of the entire project can be found in the final project report and other related publications [12-16]

### Data collection

**Figure 1:**
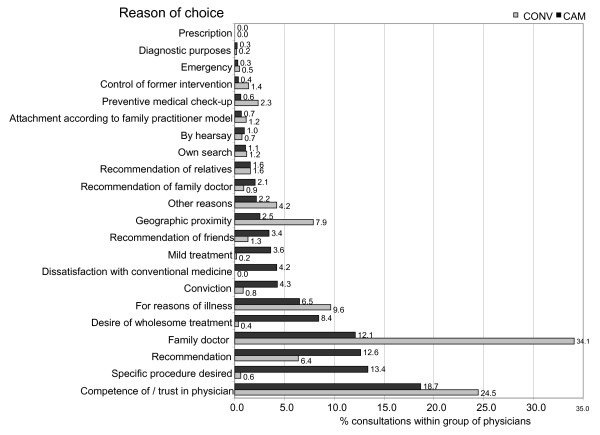
Choice of physician.

Physicians and their staff were instructed to sample all patients attending their practices on each of four given days during a 12-month period in 2002/03. Patients were asked to fill out a questionnaire prior to their consultation in a waiting room. Questions related to their socio-demographic characteristics, duration and severity of complaints, general health status, expectations, and motives for choosing a particular physician. Motives were recorded as free-text entries in a single text field. A preliminary analysis of a sub sample the data was performed in order to reduce dimensionality and to identify basic reasons and motives choice. This process was achieved inductively and yielded 22 basic reasons for choosing a particular physician, i.e. categorization was allowed to emerge from the data (figure 1). In the case of multiple disjunctive entries the first entry was selected. This classification scheme was then used to record the data in the database For interpretation and statistical analysis, these basic reasons were then further reduced into three broad categories, denoted as physician-related (competence, trustfulness of GP, etc.), procedure-related (holistic treatment, specific procedure desired, dissatisfaction with conventional medicine, etc.), or pragmatic (attachment according to family practitioner model, preventive medical check-up, geographic proximity, etc.). Reduction of dimensionality and classification of data was performed by a research group consisting of multiple physicians, a social scientist and an epidemiologist. Most text entries of the patients were equal or very similar in their wording, however, in case of uncertainty, classification was achieved after reaching consensus within the research group.

Physicians were asked to document the same consultations with reference to type of consultation, general health, severity and duration of symptoms of their patients.

All questionnaires were developed in close cooperation with an external and interdisciplinary group that included experts in conventional and complementary medicine.

Based on demographical data provided by the FMH and Santésuisse (the association of the Swiss health insurances) our data could be validated with reference to geographical distribution of practices and age and gender of patients. The ethics committee of the Canton Bern raised no objection to the study.

### Data management and data analysis

Data were recorded using a relational database. The free-text answers related to patients' main health problems were coded according to the main chapters of ICD-10. Coding was performed by two physicians and one pharmacist. Data analysis was performed with chi-square tests and multivariate regression procedures. All analytical procedures accounted for non-independence of observations at the practice level using Taylor series expansion procedures; 95% confidence intervals (95% CI) of means and proportions were calculated accordingly. The level of significance was set at p < 0.05 throughout the study.

## Results

### Study physicians

262 physicians who responded met the selection criteria and were included in the study, representing 4.3% of all Swiss primary care providers in 2002. 78 physicians (30%) were practicing conventional medicine solely (CONV) and 184 physicians (70%) were certified either in one of the alternative methods (homeopathy, 42%; anthroposophic medicine, 9%; neural therapy, 7%; traditional Chinese medicine, 20%) or had multiple CAM certificates. Among practitioners documenting five or more patients, the average number of patients registered per physician during the sampling period was 33 for CONV and 36 for CAM.

### Socio-demographic attributes of patients

Table 1 summarizes socio-demographic data of all participating patients (N = 9170). Almost one-third of all patients consulted a conventional physician, whereas two-thirds consulted a complementary physician. There were nearly six times more adults as children in the sample. The average age was slightly lower in the CAM group compared to CONV (adults: 49 and 51 years; children: 7 and 11 years, respectively). The proportion of female patients overall and of women over 16 years was significantly higher in the CAM group, whereas the percentage of girls was significantly lower in the CAM group.

**Table 1:** Socio-demographic characteristics of patients (N = 9170)

		**CONV (N = 2575)**			**CAM (N = 6595)**		
		**#**	**%**	**95% CI**	**#**	**%**	**95% CI**
Physicians	*N*	78	30		184	70	
Patients	*Children (N = 1291)*	118	9		1173	91	
	Adults* (N = 7879)	2457	31		5422	69	
	***All***	**2575**	**28**		**6595**	**72**	

Proportion of female, patients	Children**	72	61	52–70	537	46	43–48
	Adults**	1405	57	54–60	3866	71	70–73
	All*	**1477**	**57**	**54–61**	**4403**	**67**	65–68

Mean age, patients	*Children*	11 years			7 years		
	*Adults*	51 years			49 years		
	***All***	49 years			41 years		

*>16 years old

### Health status of patients

There was no significant difference in the self-perceived health status between the CONV and CAM group both among children and adult patients (results not shown).

The proportion of severe health conditions (physician-rated) was significantly higher in the CAM group for both children and adult patients (table 2). In a gender- and age-adjusted logistic regression model the proportion of chronic conditions (duration > 3 months) was found to be significantly higher in the CAM group for all patients (table 2).

**Table 2:** Patient population: general health status (physician-rated)

		**CONV**			**CAM**		
		**#**	**%**	**95% CI**	**#**	**%**	**95% CI**
General health (physician rated)	proportion of chronic conditions**						
	*Children*	19	16	13–29	415	35	41–51
	*Adults*	1067	43	52–58	3135	58	67–73
	***All***	**1086**	**42**	**50–57**	**3550**	**54**	**63–69**
	
	proportion of severe conditions**						
	*Children*	11	9	5–15	189	16	16–22
	*Adults*	476	19	19–24	1376	25	25–29
	***All***	487	19	19–24	1565	24	24–27

**Significant difference (p < 0.05) between CONV and CAM groups (age and gender adjusted analysis)

Significant differences between CAM and CONV physicians were also found with reference to the type of consultations: As many as 7% of consultations were classified as emergencies by CAM physicians vs. 11% in CONV, and 6% of consultations with CAM physicians were related to accidents vs. 8% in CONV.

### Main health problems patients attended their physicians for (based on patient data)

**Figure 2:**
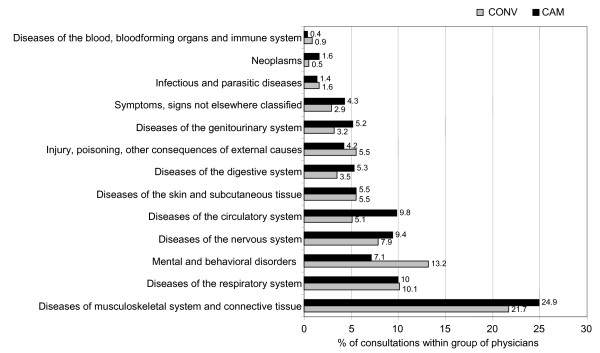
Main health reasons adults attended their physicians for.

The majority of adult CAM patients visited a physician for musculoskeletal (25%, most notably back pain), respiratory (10%, acute upper respiratory tract infection, asthma, chronic sinusitis, bronchitis, and allergic rhinopathy), nervous system (10%, migraine, multiple sclerosis, and sleep disorders) or mental and behavioural problems (9%, most frequently depression). The details are shown in figure 2.

In CONV patients musculoskeletal problems (22%) were also most common, followed by the diseases of the circulatory (13%) and then respiratory (10%) systems.

**Figure 3:**
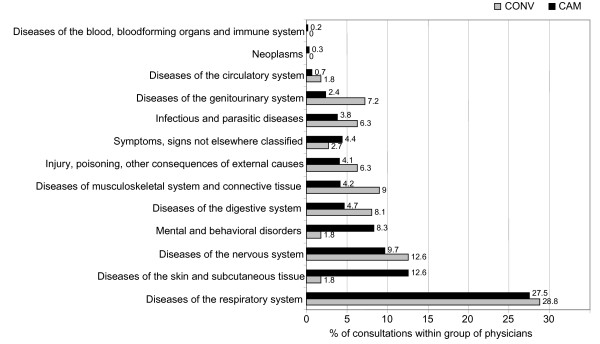
Main health reasons children attended their physicians for.

Figure 3 shows the main reasons for consultations for parents seeking care for their children. Respiratory problems were the leading issue among children. There was little difference between CAM (27%) and CONV (29%) groups. Significant differences in favour of complementary medicine were detected for diseases of the skin – (CAM 13%, CONV 2%; principally dermatitis) and mental and behavioural disorders (CAM 8%, CONV 2%; ADHD, followed by personality disorders, behavioural disorders and anxiety disorders:).

### Patients reasons for choice and expectations

Patients and parents cited 22 basic reasons for physician visits (figure 1), which fell into three broad categories, denoted as physician-related (competence, trustfulness of GP, etc.), procedure-related (holistic treatment, specific procedure desired, dissatisfaction with conventional medicine, etc.), or pragmatic (preventive medical check-up, geographic proximity, etc.).

Adult CAM patients gave the following reasons for choosing their complementary physician: relying upon recommendations of their family and friends, desire for specific procedures, holistic treatment, mild treatment, or dissatisfaction with conventional medicine. CAM patients significantly more frequently cited a preference for certain procedures, whereas physician-related and pragmatic reasons were more often mentioned among CONV-patients (table 3). Significantly more CAM patients expected healing, alleviation, mild treatment, fewer side-effects and/or lower costs. The largest difference was found with respect to adverse treatment effects: CAM patients and parents expected them less often (table 4).

**Table 3:** Choice of physician, summarized in a 3-level classification

	**CONV**			**CAM**		
	**#**	**%**	**95% CI**	**#**	**%**	**95% CI**
*Physician-related reasons (competence of/trust in physician; recommendation etc).*						
*Children*	40	33	25–43	408	35	31–38
Adults*^b^	873	35	32–39	2189	40	38–43
All*	913	35	32–38	2597	39	37–41

*Procedure-related reasons (specific procedure desired, wholesome treatment, mild treatment)*						
*Children*	-	-	-	538	46	41–50
Adults*^b^	56	2	1.6–3	1716	32	29–34
All*	56	2	1.5–3	2254	34	31–37

*Pragmatic/other reasons (attachment according to family practitioner model, geographic proximity etc)*						
*Children*	78	66	57–75	227	19	15–24
Adults*^b^	1528	62	59–65	1517	28	25–31
All*	1606	62	59–66	1744	26	23–30

* Significant difference (p < 0.05) between CONV and CAM groups (bivariate analysis)

^b^Significant difference (p < 0.05) to CONV-group in a multivariate linear or logistic model (age and gender controlled) for all female patients but for male patients between 30–65 years of age only.

**Table 4:** Patients expectations

	**CONV**			**CAM**		
Expectations (patient-rated)	**#**	**%**	**95% CI**	**#**	**%**	**95% CI**
*Healing*						
Children*	71	60	51–69	853	73	70–75
Adults*	1248	53	50–55	3112	57	56–59
All*	1369	53	51–56	3965	60	58–62

*Symptom alleviation*						
*Children*	27	23	16–30	357	30	27–33
Adults*	984	40	37–43	2420	45	43–47
All*	1011	40	36–42	2777	42	40–44

*Milder treatment*						
*Children*	13	11	5–17	177	15	13–17
Adults*	112	5	3–6	487	9	8–10
All*	125	5	4–6	664	10	9–11

*Fewer adverse side-effects*						
*Children*	9	8	2–13	280	24	21–27
*Adults*	212	8	7–10	1221	23	21–24
All*	221	9	7–10.	1501	23	21–24

*Lower costs*						
*Children*	0			50	4	3–5
*Adults*	34	1.4	0.7–2	257	5	4–5
All*	34	1.3	0.7–2	307	5	4–5

* Significant difference (p < 0.05) between CONV and CAM groups (bivariate analysis)

Children were treated by complementary physicians most often because a specific procedure was desired by their parents, followed by the parents' belief and trust in the competence of the physician, and by their preference for a comprehensive treatment. About one-third of the parents made their choice due to physician-related reasons (in both groups, table 3). Considerable differences between groups were found with regard to procedure-related and pragmatic reasons. While 46% of parents in the CAM group chose their physicians on the basis of procedure-related grounds, none did so in the CONV group. CONV patients more often cited pragmatic reasons (66%).

## Discussion

### Sample characteristics

Socio-demographic variables and current health status of patients distinguished CAM users and non-users [12]. Though several authors[7,17] argued that neither sex nor age predicted CAM use, we found a significantly higher percentage of women in among the CAM group, which is in accord with the U.S[18,19]. In our sample, CAM patients were younger (in both children and adult groups). One of the most surprising results was that in spite of a significantly higher proportion of chronic and/or severe conditions among CAM users (consistent with [19], no significant differences in the patients self-perceived health status were found (in contrast to results of [8,19,20].

### Patients' reasons for choice of physician and expectations

Substantial difference between two groups was found in terms of procedure-related motives. CAM patients stated that a preference for a specific procedure, desire of a comprehensive/mild treatment, and their personal conviction were of great importance to them. At the same time, very few CONV patients mentioned those reasons.

Twice as many CAM patients (compared to CONV) chose their practitioner because of recommendations. Parents' reasons for choices on behalf of their children were similar to those of adult patients.

There are currently three main reasons explaining patients' choice of alternative medicine[7].

1. Dissatisfaction with orthodox medicine: necessity to treat conditions unresponsive to conventional treatments and/or negative past experiences with conventional medical services. This theory implies that people are choosing alternative health care for expedient reasons: CAM methods are perceived not only as effective, but also as milder and causing less adverse side-effects [1,17,21].

2. Determination for more personal involvement in the healing process in order to keep control over own health care decisions. This may result not only in sole preference for CAM, but also in a choosing of combined use of CAM and CONV methods [7,22].

3. Philosophical compatibility: CAM therapies are attractive because they are perceived as more congruent with patients' spiritual/religious values, beliefs or philosophy regarding the nature and meaning of health and illness [23-25].

The first theory does not account for all patient choices but plays a certain role along with the other two [26]. The latter two are not related to clinical success and are often associated with globalization, and include more sophisticated consumer choice and increased competition among health care providers. Such competition leads, in turn, first to a power shift from provider to consumer, and then to commercialization of values and tradition [26]. This raises a question: should the application of public funds be directed by consumer demand? The population of CAM patients apparently uses health care resources more frequently [27] and in a more diverse way [12,17] and it cannot be excluded that this behaviour is related to the fact that these patients have more often a specific procedure in mind when they seek a physician.

The fact that CAM users less often cite pragmatic reasons for seeing a physician may also be related to the observation that CAM physicians care for only a specific subset of patients in primary care [28]; that is, they provide significantly less emergency care and have fewer accident patients, and less often make home visits – a pattern not fully in line with the general definition of general practice/family medicine [29]. Furthermore, other data within the main project showed considerable differences between physicians for the self declared extent of medical activity in primary care where CONV physicians declared 77.4% (median 90%) of their activity as primary care and CAM physicians only 36.8% (39%)[14]. The observed differences in reasons of choosing a particular physician may therefore not only be related to distinct differences in the decision-making process of patients[12] but also to attributes of physicians themselves.

From a health system perspective, however, our results have several implications:

- There may be a downside to boundless shopping around for physicians and procedures. There are inverse relationships between patient empowerment and cost effectiveness in health care [4]. Limiting the choice of patients in managed care practices, for instance, is associated with reduction of health care costs while quality of outcomes are maintained [30,31]. It may be argued in this context, that CAM provides more efficient care than CONV as patient satisfaction in CAM is higher and cost appear to be equal to CONV[13,14,32]. However, this gain of efficiency may by compensated at system level by the fact that CAM patients tend to utilize health related resources more frequently than CONV patients[14].

- The obvious mismatch of defined and self-concept of practice activity may adversely affect decisions on resource allocation and reimbursement policy for CAM in primary care.

### Limitations and strengths

This analysis is only one part of a larger study of alternative medicine in Switzerland, and therefore may suffer from several limitations and caveats common for this type of research. The questionnaires did not allow for an in-depth assessment of absolutely all aspects of the patients motivation, and due to the requirements of statistical analysis the broad variety of motivation was reduced to a few coding categories. Such categories may not reflect the diversity of views and motivations of patients, which may be grounded in different philosophical traditions. CAM was evaluated as an undifferentiated whole; no attempt was made to distinguish between various types of alternative medicine practices (for example, motives of patients attending a traditional Chinese medicine practitioner could differ from those attending a homeopath). A further problem in this context is related to the rationale of using only the first entry in the questionnaire as the motive to consult a specific physician. However, the text field in the questionnaire provided only little room for handwritten entries and multiple motives of consultations were consequently very rarely given by patients. It is therefore unlikely that differences in patient's motives between groups were affected by this restriction. Additional limitations in this context refer to the fact that only CAM procedures provided by certified physicians were included in the study. However, the evaluation of CAM provided by other care providers or self-care CAM or CAM was not in the scope of the main project.

Low response was a problem in this study as physicians perceived the entire project as a government project[15], which led to some reservations to participate. A formal evaluation of the proportion of participating physicians could not be performed due to the fact the proportion of physicians providing CAM procedures without corresponding certification was not known prior to the study. It is therefore also not possible to calculate the sampling fraction of physicians performing no CAM procedures at all (CONV group). However, it can be assumed that the motivation among participating physicians was different, since CAM physicians were under pressure to demonstrate effective methods – which was not the case for CONV physicians. It can only be speculated that the motivation of CONV physicians is more attributable to a general interest in primary care research. In a strict sense, the generalisability of our results is therefore reduced to physicians with these distinct motivations.

Health insurer data, information of the Swiss medical association and data from other recent studies in Swiss primary care[16,33,34] were used to check our data for potential biases. Based on this additional information, we have no reason to consider our sample as well as our results as biased with regard to geographical distribution and gender of physicians and to health status of patients.

Nevertheless, this is the first study of its type in the country, with substantial sample size and sufficient time span. That is why we are reasonably sure that the results accurately describe the motivations CAM patients to consult a primary care physician in Switzerland.

## Conclusion

The study findings may serve to better the understanding of patients' needs in shaping future health care policies and for promoting mutually beneficial integration of conventional and complementary medicine. Growth of societal awareness and willingness to assume more control over personal health care decisions reflects an ongoing shift in the interrelationship between availability and utilisation of medical resources in populations with practically non-restricted access to health services. A substantial number of patients tend to choose their CAM practitioner out of a wish for a specific procedure (unrelated to clinical success). With all due respect to personal right to participate actively in the healing process, patient-cantered medical practice is not reducible to mere fulfilment of every patients' desire. Another crucial task of the care providers is to improve clinical success while keeping costs under control.

## Competing interests

The author(s) declare that they have no competing interests.

## Authors' contributions

VW wrote the manuscript. AB obtained the mandate to perform the study; he set up the database, performed all statistical analyses and reviewed and completed the manuscript. Both authors read and approved the final version of the manuscript.

## Pre-publication history

The pre-publication history for this paper can be accessed here:



## References

[B1] Bensoussan A (1999). Complementary medicine – where lies its appeal?. MJA.

[B2] Fisher P, Ward A (1994). Complementary medicine in Europe. BMJ.

[B3] MacLennan AH, Wilson DH, Taylor AW (1996). Prevalence and cost of alternative medicine in Australia. Lancet.

[B4] MacLennan AH, Wilson DH, Taylor AW (2002). The escalating cost and prevalence of alternative medicine. Prev Med.

[B5] Schar A, Messerli-Rohrbach V (1999). [Motivation for the choice of complementary and mainstream medicine. Patients' behavior in a pluralistic medical system]. Forsch Komplementarmed.

[B6] Schar A, Messerli-Rohrbach V, Schubarth P (1994). Conventional or complementary medicine: what criteria for choosing do patients use?. 1: Schweiz Med Wochenschr.

[B7] Astin JA (1998). Why patients use alternative medicine: results of a national study. JAMA.

[B8] Wolsko P, Ware L, Kutner J, Lin CT, Albertson G, Cyran L, Schilling L, Anderson RJ (2000). Alternative/complementary medicine: wider usage than generally appreciated. J Altern Complement Med.

[B9] Harris KM (2003). How do patients choose physicians? Evidence from a national survey of enrollees in employment-related health plans. Health Serv Res.

[B10] Cincotta DR, Crawford NW, Lim A, Cranswick NE, Skull S, South M, Powell CV (2006). Comparison of complementary and alternative medicine use: reasons and motivations between two tertiary children's hospitals. Arch Dis Child.

[B11] Hall JD, Bissonette EA, Boyd JC, Theodorescu D (2003). Motivations and influences on the use of complementary medicine in patients with localized prostate cancer treated with curative intent: results of a pilot study. BJU Int.

[B12] Busato A, Donges A, Herren S, Widmer M, Marian F (2005). Health status and health care utilisation of patients in complementary and conventional primary care in Switzerland – an observational study. Fam Pract.

[B13] Melchart D, Mitscherlich F, Amiet M, Eichenberger R, Koch P (2005). Programm Evaluation Komplementärmedizin (PEK) Schlussbericht.

[B14] Busato A, Eichenberger R, Kuenzi B (2006). Extent and structure of health insurance expenditures for complementary and alternative medicine in Swiss primary care. BMC Health Serv Res.

[B15] Walach H, Linde K, Eichenberger R, Stalder H, Kristensen FB, Kleijnen J (2006). Summary consensus statement of the PEK review board regarding the PEK process and the PEK products. J Altern Complement Med.

[B16] Widmer M, Herren S, Donges A, Marian F, Busato A (2006). Complementary and conventional medicine in Switzerland: comparing characteristics of general practitioners. Forsch Komplementarmed.

[B17] Furnham A, Forey J (1994). The attitudes, behaviors and beliefs of patients of conventional vs. complementary (alternative) medicine. J Clin Psychol.

[B18] Tindle HA, Davis RB, Phillips RS, Eisenberg DM (2005). Trends in use of complementary and alternative medicine by US adults: 1997–2002. Altern Ther Health Med.

[B19] Eisenberg DM, Davis RB, Ettner SL, Appel S, Wilkey S, Van Rompay M, Kessler RC, Thomas KJ, Nicholl JP, Coleman P (1998). Trends in alternative medicine use in the United States, 1990–1997: results of a follow-up national survey. Use and expenditure on complementary medicine in England: a population based survey. Surveys of complementary and alternative medicine: part I. General trends and demographic groups. JAMA.

[B20] Ramos-Remus C, Watters CA, Dyke L, Suarez-Almazor ME, Russell AS (1999). Assessment of health locus of control in the use of nonconventional remedies by patients with rheumatic diseases. J Rheumatol.

[B21] Vincent C, Furnham A (1996). Why do patients turn to complementary medicine? An empirical study. Br J Clin Psychol.

[B22] Sirois FM, Gick ML (2002). An investigation of the health beliefs and motivations of complementary medicine clients. Soc Sci Med.

[B23] Caspi O, Koithan M, Criddle MW (2004). Alternative medicine or "alternative" patients: a qualitative study of patient-oriented decision-making processes with respect to complementary and alternative medicine. Med Decis Making.

[B24] Bussing A, Matthiessen PF, Ostermann T (2005). Engagement of patients in religious and spiritual practices: confirmatory results with the SpREUK-P 1.1 questionnaire as a tool of quality of life research. Health Qual Life Outcomes.

[B25] Zollman C, Vickers A (1999). ABC of complementary medicine. Complementary medicine in conventional practice. Bmj.

[B26] Eastwood H (2000). Complementary therapies: the appeal to general practitioners. Medical Journal of Australia.

[B27] Eisenberg DM, Kessler RC, Foster C, Norlock FE, Calkins DR, Delbanco TL (1993). Unconventional medicine in the United States. Prevalence, costs, and patterns of use. N Engl J Med.

[B28] Blaise R, Maiga A, Aboubacar A (1997). How different are users and non-users of alternative medicine?. Can J Pub Health.

[B29] (2002). WONCA Europe. http://www.woncaeurope.org/Definition%20GP-FM.htm.

[B30] Luft HS (1995). HMOs, market competition, and premium cost. Journal of Health Economics.

[B31] Feuz P (2006). Viel günstiger und trotzdem noch gut. Der Bund.

[B32] Witt C, Keil T, Selim D, Roll S, Vance W, Wegscheider K, Willich SN (2005). Outcome and costs of homoeopathic and conventional treatment strategies: a comparative cohort study in patients with chronic disorders. Complement Ther Med.

[B33] Künzi B (2004). Swisspep Qualidoc® gibt Rechenschaft über hausärztliche Wirksamkeit Zürich.

[B34] Künzi B (2004). Swisspep Qualidoc®: A balanced score card to capture and extend the added values of general practice/family medicine.

